# Defective fibrin deposition and thrombus stability in *Bambi* ^−/−^ mice are mediated by elevated anticoagulant function

**DOI:** 10.1111/jth.14593

**Published:** 2019-08-26

**Authors:** James T. B. Crawley, Argita Zalli, James H. Monkman, Anastasis Petri, David A. Lane, Josefin Ahnstrӧm, Isabelle I. Salles‐Crawley

**Affiliations:** ^1^ Centre for Haematology Hammersmith Hospital Campus Imperial College London London UK

**Keywords:** anticoagulants, endothelial cells, fibrin, mice, thrombomodulin, thrombosis

## Abstract

**Background:**

Bone morphogenetic and activin membrane‐bound inhibitor (BAMBI) is a transmembrane protein related to the type I transforming growth factor‐ β (TGF‐β) receptor family that is present on both platelets and endothelial cells (ECs). *Bambi*‐deficient mice exhibit reduced hemostatic function and thrombus stability characterized by an increased embolization.

**Objective:**

We aimed to delineate how BAMBI influences endothelial function and thrombus stability.

**Methods:**

*Bambi*‐deficient mice were subjected to the laser‐induced thrombosis model where platelet and fibrin accumulation was evaluated. Expression of thrombomodulin and tissue factor pathway inhibitor (TFPI) was also assessed in these mice.

**Results:**

Thrombus instability in *Bambi* ^−/−^ mice was associated with a profound defect in fibrin deposition. Injection of hirudin into *Bambi* ^+/+^ mice prior to thrombus formation recapitulated the *Bambi* ^−/−^ thrombus instability phenotype. In contrast, hirudin had no additional effect upon thrombus formation in *Bambi* ^−/−^ mice. Deletion of *Bambi* in ECs resulted in mice with defective thrombus stability caused by decreased fibrin accumulation. Increased levels of the anticoagulant proteins TFPI and thrombomodulin were detected in *Bambi* ^−/−^ mouse lung homogenates. Endothelial cells isolated from *Bambi* ^−/−^ mouse lungs exhibited enhanced ability to activate protein C due to elevated thrombomodulin levels. Blocking thrombomodulin and TFPI in vivo fully restored fibrin accumulation and thrombus stability in *Bambi* ^−/−^mice.

**Conclusions:**

We demonstrate that endothelial BAMBI influences fibrin generation and thrombus stability by modulating thrombomodulin and TFPI anticoagulant function of the endothelium; we also highlight the importance of these anticoagulant proteins in the laser‐induced thrombosis model.

**Essentials**

- BAMBI is a transmembrane protein highly expressed in platelets and endothelial cells.
- Endothelial BAMBI deficiency reduces fibrin accumulation and increases thrombus instability.
- The thromboprotective effect is manifest by TFPI and thrombomodulin functions on the endothelium.
- TFPI plays an important role in fibrin accumulation in the laser‐induced thrombosis model.

## 1 INTRODUCTION

Hemostatic plug formation after vessel injury serves to limit blood loss and maintain vascular integrity. It involves concerted roles of the vessel wall, platelets, and other blood cells (e.g. neutrophils, red blood cells), as well as the coagulation cascade.^1^, ^2^, ^3^, ^4^, ^5^, ^6^ Using experimental mouse models of thrombosis, or human blood in ex vivo flow assays, previous studies have shown that thrombi exhibit a heterogeneous composition with a core of closely packed, activated platelets that is overlaid by a shell of less‐activated platelets.^3^, ^7^ The core of the thrombus (compared to the shell) has reduced porosity and solute transport that effectively increases the local thrombin concentration leading to more efficient fibrin deposition and thrombus stabilization.^8^, ^9^ Many proteins expressed in the endothelium, platelets, and plasma influence both the formation and stabilization of a thrombus.^10^

Endothelial cells play a critical role in regulating hemostatic plug formation through production of a variety of thromboprotective agents. These include vasodilators, such as nitric oxide and prostacyclin, that also influence platelet function, as well as anticoagulant proteins such as TFPI and thrombomodulin. The TFPI is a Kunitz‐type serine protease inhibitor that, together with its cofactor protein S, targets the initiation phase of coagulation by direct inhibition of tissue factor‐factor VIIa (TF‐FVIIa) and FXa.^11^, ^12^, ^13^ TFPI exists in two alternatively spliced isoforms, TFPIα and TFPIβ, which are both expressed in EC. In humans, full‐length TFPIα is primarily secreted into the plasma, where it circulates at low concentrations (0.1‐0.2 nM). Conversely, the shorter TFPIβ remains GPI‐linked to the endothelial cell surface.^14^ Both isoforms inhibit TF‐FVIIa in a FXa‐dependent manner, with TFPIα likely being more important for regulating hemostasis on platelet surfaces, whereas TFPIβ probably functions primarily on the endothelial cell surface to which it is attached.^15^ In mice, a shorter soluble TFPI isoform (TFPIγ) consisting of the first two Kunitz domains also exists in circulation.^16^, ^17^, ^18^ Thrombomodulin is a type‐I transmembrane protein expressed by EC.^19^ Thrombomodulin binds thrombin with high affinity and enhances the conversion of protein C into activated protein C (APC) by ~1000‐fold.^20^ In larger vessels, endothelial cell protein C receptor colocalizes with thrombomodulin in lipid rafts and further enhances APC generation (~20 times) by the thrombin‐thrombomodulin complex.^21^, ^22^ Once activated, APC proteolytically inactivates FVa and FVIIIa, thereby regulating the propagation phase of coagulation. Modulation of APC activity has recently been shown to influence thrombus formation in a hemophilic mouse model.^23^

Bone morphogenetic protein (BMP) and activin membrane‐bound inhibitor (BAMBI) is a 260‐amino‐acid transmembrane protein that has been assigned to the TGF‐β superfamily because of the high homology of its extracellular domain to the TGF‐β type I receptors (TGF‐βRI).^24^ It has been postulated that BAMBI antagonizes TGF‐β/BMP/activin signaling by preventing the formation of TGF‐β type I/II receptor heterocomplexes, although the molecular basis of its function remains unclear.^24^, ^25^, ^26^ Interestingly, BAMBI lacks the intracellular kinase domain normally present in TGF‐βRI. Furthermore, its intracellular domain shares no homology to any other protein or domain, making it difficult to predict the function or mode of action of this domain. In vertebrates BAMBI is highly conserved,^24^, ^27^, ^28^, ^29^ and its transcript is highly expressed in EC and megakaryocytes when compared to other blood cell lineages.^30^, ^31^ *Bambi*‐deficient mice are viable and fertile,^32^, ^33^, ^34^ but we have previously reported a proportion of *Bambi* ^−/−^ pups dying around weaning age.^34^ *Bambi* ^−/−^ mice exhibit enhanced angiogenesis in vivo and are more susceptible to diabetic glomerular disease, where an important role of BAMBI in the endothelium was noted.^35^, ^36^ We recently demonstrated that BAMBI acts as a positive regulator of thrombus formation and stability and plays a role in hemostasis.^34^ BAMBI deficiency had no effect on platelet count, ex vivo platelet activation, aggregation or procoagulant function, and no influence on the endogenous thrombin potential of plasma. Thrombus formation in *Bambi* chimeric mice revealed that BAMBI in the vessel wall (rather than in the hematopoietic compartment) was important for thrombus stability.^34^ In the present study, we define the mechanisms by which BAMBI in the endothelium (rather than in blood cells, platelets, or extravascular locations) exerts its function and how these influence thrombus formation and stability.

## 2 METHODS

### 2.1 Generation of Bambi^flox/flox^ Tie2‐Cre^+^ mice

All animal work was performed in compliance with Imperial College animal ethics guidelines according to the UK Home Office's Animals (Scientific Procedures) Act 1986. The *Bambi* ^flox/flox^ and *Bambi* ^−/−^ mice backcrossed on a C57BL/6 background (>10 generations) have been described previously.^32^, ^34^ To generate endothelial *Bambi* knock‐out mice, *Tie2‐Cre* [Jackson Laboratory no 008863 B6.Cg‐Tg(Tek‐cre)1Ywa/J] males were crossed with *Bambi* ^flox/flox^ female mice. *Bambi* ^flox/+^ *Tie2‐Cre* ^*+*^ males were further bred with *Bambi* ^flox/flox^ female mice to generate *Bambi* ^flox/flox^ *Tie2‐Cre* ^+^ mice. Genotyping of *Bambi* ^flox/flox^ and *Bambi* ^−/−^ have been previously described.^32^, ^34^ For the detection of the *Cre* allele the following primers (FW: GCCTGCATTACCGGTCGATGCAACGA and R: GTGGCAGATGGCGCGGCAACACCATT) were used.

### 2.2 Prostacyclin and nitric oxide plasma levels

Prostacyclin plasma levels were determined by measuring the stable analogue 6‐keto‐PGF_1α_ by enzyme‐linked immunosorbent assay according to the manufacturer's specifications (Cayman Chemical). Plasma nitrite and nitrate levels were determined by Griess colorimetric assay according to the manufacturer's instructions (Cayman Chemical).

### 2.3 Isolation of MLEC and MBEC

Primary mouse lung endothelial cells (MLEC) were isolated as previously described with modifications.^37^ Briefly, minced lungs from <3‐week‐old mice were digested with 0.2% collagenase type II (Invitrogen, Paisley, UK). Cells were sieved (70‐μm cell strainer, Corning, Amsterdam, The Netherlands) and centrifuged at 350[qqqmulti sign]x*g* for 5 min and resuspended in 1 mL of isolation medium. Endothelial cells were selected by immunosorting using sheep antirat Dynabeads (Invitrogen) precoated with rat antimouse platelet endothelial cell adhesion molecule 1 (PECAM‐1) antibody (MEC 13.3, BD Biosciences, Wokingham, UK). Bound EC were released from the beads using trypsin digestion. Cells were cultured in Microvascular Endothelial Growth Medium‐2 (EGM‐2 MV media; Lonza, Basel, Switzerland) in 6‐well plates (1 well/mouse) precoated with 1× Attachment Factor Protein (Invitrogen). Purity of the cells was assessed by flow cytometry using rat antimouse CD54‐Alexa 488, rat antimouse CD31‐PE/Cy7, rat antimouse CD102‐Alexa 647, and corresponding control immunoglobulin G (IgG) antibodies (Biolegend, London, UK). The MLECs (purity 60%‐90%) were passaged two to eight times and used for all experiments.

Primary mouse brain endothelial cells (MBECs) were isolated as previously described.^38^ Briefly, brains from five sex‐matched and age‐matched *Bambi* ^−/−^ and *Bambi* ^+/+^ mice were rolled on a sterile filter paper to remove meninges. Brains were transferred into a hand homogenizer with isolation medium: Hanks buffered salt solution (Invitrogen) supplemented with 10 mM 4‐(2‐hydroxyethyl)‐1‐piperazineethanesulfonic acid, 1× penicillin‐streptomycin (Sigma, Gillingham, UK) and 0.5% BSA (First Link, Birmingham, UK). Brain microvessels were obtained by series of centrifugation in 22% bovine serum albuminBSA, and digested in 0.5 mg/ml collagenase/dispase (Roche, Burgess Hill, UK) and 0.5 mg/ml collagenase (Invitrogen) for 60‐90 min at 37°C. Cells were centrifuged, washed and resuspended in EGM‐2 media supplemented with growth factors (CC‐4176; Lonza) and 5 μg/ml puromycin (first 48 h only). Cells were grown to confluency in fibronectin (Sigma) coated wells.

### 2.4 Western blot

Whole cell protein lysates were prepared from lungs, MLEC and MBEC with RIPA buffer (Sigma) supplemented with protease and phosphatase inhibitors (cOmplete, mini and PhosSTOP from Roche). Protein concentrations were determined with BCA protein assay (Thermo Fisher Scientific, Loughborough, UK). Immunoblotting of lysates was performed using reducing conditions (except for TFPI expression levels) with 4‐12% NuPAGE pre‐cast gels and transfer units (Invitrogen). Blocked membranes were incubated overnight at 4°C with the following primary antibodies: mouse anti‐porcine Glyceraldehyde 3‐phosphate dehydrogenase (GAPDH) (Novus Biological, Cambridge, UK), rabbit anti‐mouse eNOS and peNOS (Santa Cruz Biotechnology, Heidelberg, Germany), rat anti‐mouse PECAM‐1 (BD Biosciences), rat anti‐mouse ICAM‐1 (Biolegend), goat anti‐mouse thrombomodulin (R&D, Bio‐Techne, Abingdon, UK), goat anti‐mouse TFPI (R&D, Bio‐Techne), rabbit anti‐human fibrinogen α (Santa Cruz Biotechnology). Detection and quantification of chemiluminescence intensity were quantified by using Chemidoc^TM^ imaging system and Image Lab 5.2.1 software (Biorad, Watford, UK). Protein levels were quantified and normalised against loading controls (GAPDH).

### 2.5 Activated protein CPCgeneration assay

APC generation assays were carried out as previously described.^39^ MLEC from *Bambi* ^−/−^ mice and wild‐type littermates were isolated in parallel and grown to confluency. Cells were washed twice in Hanks buffered salt solutionHBSS and once in assay buffer (20 mM Tris, 100 mM NaCl, 1 mM CaCl_2_, 0.1% bovine serum albumin pH 7.5) prior to addition of 100 nM recombinant human protein C (see later discussion) together with 2 nM human thrombin (Enzyme Research Laboratories, Swansea, UK). The APC activation occurred at 37 °C and reactions were stopped (0‐60 min) by addition of hirudin (100 nM; Sigma). Control cells were preincubated with 50 nM goat anti‐ thrombomodulin (R&D, Bio‐Techne) antibody for 30 min to block endogenous thrombomodulin and washed three times before addition of protein C. The APC was quantified by cleavage of the chromogenic substrate S‐2366 (0.5 mM; Quadratech Diagnostics for Chromogenix, Epsom, UK). The rate of cleavage was followed for 20 min at 37 °C using an EL x808 plate reader (Perkin Elmer) and the concentration of APC extrapolated from a standard curve of purified human APC (Cambridge Bioscience for Haematologic Technologies Inc., Cambridge, UK). Control experiments lacking thrombin or protein C were performed to demonstrate the specificity of the substrate cleavage.

### 2.6 Protein C expression and purification

Human protein C was expressed using stably transfected HEK293 cells, as previously described.^40^ Vitamin K was added to culture medium to enable γ‐carboxylation. Conditioned media containing protein C were harvested and concentrated ~20‐fold by tangential flow filtration (Millipore, Watford, UK). Protein C was purified by barium citrate precipitation, according to a previously described protocol,^41^ followed by anion‐exchange chromatography. For this, the partially purified protein C was applied on a HiTrap DEAE fast flow column (5 mL, Thermo Fisher Scientific for GE Healthcare) equilibrated with Tris buffered solution (TBS). After washing with the same buffer, protein C was eluted with a 35 ‐mL linear CaCl_2_ gradient (0‐30 mM). Fractions were analyzed using 4% to 15% sodium dodecyl sulfate‐polyacrylamide gel electrophoresis (SDS‐PAGE) under nonreducing conditions and those containing pure protein C were pooled and dialyzed in TBS, 3 mM CaCl_2_. The pure protein C was concentrated, as required, and the protein C concentration determined by absorbance at 280 nm using extinction coefficient (E1%, 1 cm) of 14.5.^42^

### 2.7 Calibrated automated thrombography

Thrombin generation was assessed by calibrated automated thrombography (CAT) using a Fluoroskan Ascent FL plate reader (Thermo, Paisley, UK) in combination with Thrombinoscope software (Synapse BV) as described previously.^13^, ^43^ Briefly, the generation of thrombin was quantified in human plasma (80 μL per well) supplemented with 4 μM phospholipid vesicles in the presence and absence of 10 nM recombinant mouse thrombomodulin (R&D, Bio‐Techne) and 10 to 100 nM goat antimouse thrombomodulin antibody (R&D, Bio‐Techne). Coagulation was initiated with 4pM TF (Dade Innovin, Dade Behring, Camberley, UK) and 16.6 mM CaCl_2_. Contact activation coagulation was inhibited by adding corn trypsin inhibitor (40 μg/mL plasma) and thrombin generation quantified by adding 0.42 mM of Z‐GlyArg‐AMC‐HCl (Cambridge Bioscience for Bachem, Cambridge, UK).

### 2.8 Factor Xa inhibition assay by TFPI

Factor Xa (0.5 nM; Enzyme Research Laboratories) activity was monitored by the cleavage of the chromogenic substrate S‐2765 (200 μM; Quadratech Diagnostics for Chromogenix) for 40 min at 25 °C in the presence or absence of 8 nM recombinant murine TFPI (mTFPI), 40 nM polyclonal goat antimouse TFPI (R&D, Bio‐Techne) in the presence of 25 μM phospohlipids and 5 mM CaCl_2_, as described previously.^12^, ^13^

### 2.9 Intravital microscopy

The laser‐induced thrombosis model was performed as previously described using a VIVO platform (3i, London,UK).^34^ Briefly, mice were anaesthetized with ketamine (75 mg/kg) and medetomidine (1 mg/kg) and given additional ketamine (7.5 mg/kg) every 40 min to maintain anaesthesia. Platelets, neutrophil, or fibrin was visualized by injecting Dy‐Light 488 conjugated rat anti‐mouse glycoprotein Iβ (GPIβ) (0.15 μg/g; Emfret, Eibelstadt, Germany), phycoerythrin (PE) conjugated rat antimouse Ly6G (0.15 μg/g; BD Biosciences), or Alexa 647 conjugated fibrinogen (5% total fibrinogen; Invitrogen), respectively, 15 min prior to the laser injury via a cannula placed in the jugular vein. Control experiments using labeled control IgG/proteins were carried out to ensure detection of specific fluorescence in Alexa‐488, PE‐ or Alexa‐647 channels. Goat antimouse thrombomodulin or antimouse TFPI antibodies or goat IgG (R&D, Bio‐Techne; 1.9 μg/g) were injected together with reagents labeling platelets and fibrin. When indicated, hirudin (Refludan, CSL Behring GmbH, 15 μg/g) was injected into mice after four or five thrombi. The vessel wall injury was performed by a pulse laser (Ablate!, 3i) focused through a 63× water‐immersion objective (65%‐75% intensity, 5‐15 pulses). Image analysis was performed as previously described to determine the median integrated fluorescence intensity over time; differences in platelet or fibrin accumulation between genotypes were assessed by comparing the median values of area under the curves for fluorescence at 490 nm and 647 nm, respectively, versus time. 34 Thrombus embolization events were assessed by counting the number of emboli (>25% maximum thrombus size) detaching from the injury site/thrombus over time. Genotypes of the mice were blinded to the operator during data acquisition and analysis.

### 2.10 Statistical analysis

Unless otherwise indicated, results are presented as mean ± SEM from *n* ≥ 3 mice per experimental group and analyzed using GraphPad Prism (v7). Statistical analysis was performed using the unpaired student *t* test with Welsch correction when applicable for parametric comparisons or the Mann Whitney test for nonparametric comparisons.

## 3 RESULTS

### 3.1 *Bambi* ^−/−^ mice have normal vasodilator function of the endothelium

We previously demonstrated that BAMBI in the vessel wall (rather than the hematopoietic compartment) is important for thrombus stability.^34^ As BAMBI is highly expressed in EC, we hypothesized that loss of BAMBI function may alter the hemostatic function of the endothelium. Levels of the stable derivative of prostacyclin, 6‐keto‐PGF_1α,_ were similar in plasma from *Bambi* ^−/−^ and *Bambi* ^+/+^ mice, and no differences in plasma nitrate and nitrite levels were detected, indicating normal nitric oxide production (Figure S1A,B). Moreover, there was no difference in levels of phosphorylated endothelial nitric oxide synthase in lung extracts from *Bambi*‐deficient mice (Figure S1C). Together, these results suggest that neither prostacyclin nor nitric oxide levels influence the thrombus stability in *Bambi‐*deficient mice.

### 3.2 *Bambi* deficiency does not alter neutrophil recruitment to the endothelium following laser injury

**Figure 1:**
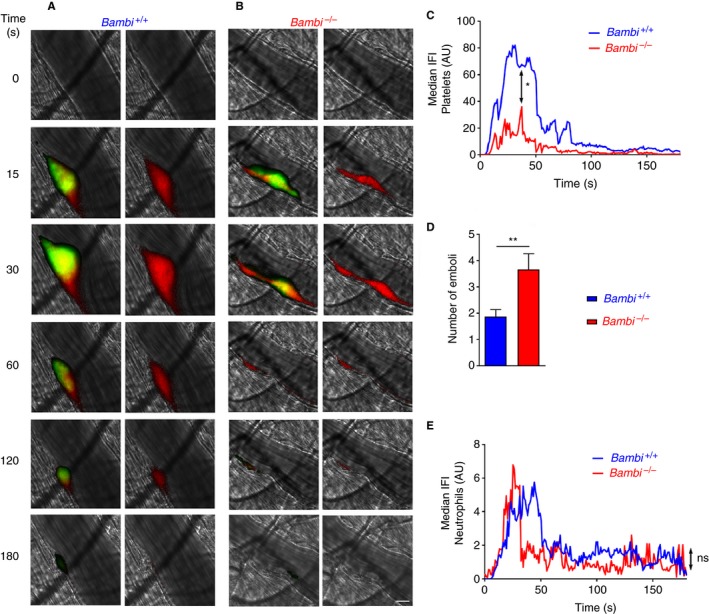
Similar kinetics of neutrophil recruitment in *Bambi*‐deficient mice after laser‐ induced thrombosis model in vivo. A, B, Representative composite fluorescence images of platelets (green) and neutrophils (red) (left panels) or neutrophils only (right panels) with bright field images after the laser injury in cremaster arterioles of A, *Bambi* ^+/+^ and B, *Bambi* ^−/−^ mice. Platelets and neutrophils were visualized by injecting rat anti‐GPIbβ‐DyLight 488 and rat anti‐Ly6G‐PE antibodies, respectively. A yellow color is seen when platelets and neutrophils are detected in the same thrombus region. Scale bar represents 10 μm. C, Graph represents median integrated fluorescence intensity (IFI) from platelets (AU, arbitrary unit) as a function of time after the injury (23 to 25 thrombi in three mice for each genotype). The area under the curve of fluorescence intensity over 180 s was analyzed using an unpaired Mann Whitney test; *P < .05. D, Number of emboli (>25% maximal thrombus size) counted during 3 min after laser injury. Results are shown as mean ± SEM. Statistical analysis was performed using unpaired student t test. **P < .01. E, Graph represents median neutrophil‐IFI (AU) as a function of time after the laser injury. The area under the curve of fluorescence intensity over 180 s was analyzed using an unpaired Mann Whitney test; ns: *P* > .05. See also Movie S1

Similar to our previous results,^34^ *Bambi* ^−/−^ mice formed thrombi, revealed by the normal time to maximal thrombus and the maximal thrombus size (Figure S2A,B). However, in stark contrast to wild‐type littermates, they were unable to sustain the thrombi at the site of injury (Movie S1, Figure 1A‐D). The overall extent of thrombus formation (i.e. area under the curve) was reduced (Figure 1A‐C; Figure S2C) and the number of embolization events (excluding normal shedding observed in this laser injury model) were significantly increased in *Bambi* ^−/−^ mice compared to *Bambi* ^+/+^ littermates (Figure 1D).

In the laser injury thrombosis model, neutrophils have been reported to interact with the activated endothelium and augment thrombus formation.^2^ In this model, blocking endothelial intercellular adhesion molecule 1 (ICAM‐1) prevents neutrophil recruitment and consequently greatly reduces both platelet and fibrin accumulation after the vascular injury.^2^ We therefore examined neutrophil accumulation in *Bambi* ^−/−^ mice using the laser‐induced thrombosis model. Interestingly, there was no difference in the kinetics of neutrophil recruitment after the injury (Figure 1E) or in the peak neutrophil accumulation (maximal integrated fluorescence intensity neutrophils; data not shown).

**Figure 2:**
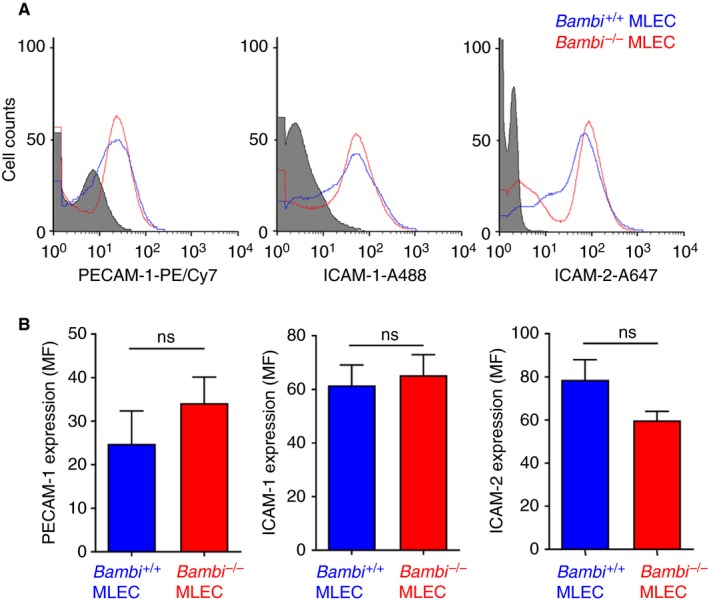
PECAM‐1, ICAM‐1, and ICAM‐2 are normally expressed in *Bambi* ^−/−^ MLEC. *Bambi* ^+/+^ MLEC and *Bambi* ^−/−^ MLEC were isolated and cultured on gelatin‐coated plates. A, Representative flow cytometry analysis of *Bambi* ^+/+^ MLEC and *Bambi* ^−/−^ MLEC for endothelial markers PECAM‐1, ICAM‐1, and ICAM‐2. B, Results are given as mean fluorescence intensities (MFI) ± SEM *(n* ≥ 10 from three separate isolations; passages 2‐ to 8). Statistical analysis was performed using unpaired Student *t* test: nonsignificant (ns), *P* > .05. ICAM, intercellular adhesion molecule; MLEC, mouse lung endothelial cell; PECAM, platelet endothelial cell adhesion molecule

Consistent with these in vivo observations, MLEC isolated from *Bambi* ^−/−^ mice displayed broadly similar expression levels of ICAM‐1 (also other endothelial cell adhesion molecules, PECAM‐1 and ICAM‐2) (Figure 2), suggesting that neutrophils bind normally to the activated endothelium after laser injury and do not impact upon thrombus stability in *Bambi*‐deficient mice.

### 3.3 Thrombus instability in Bambi^−/−^ mice is accompanied by lack of fibrin accumulation

**Figure 3:**
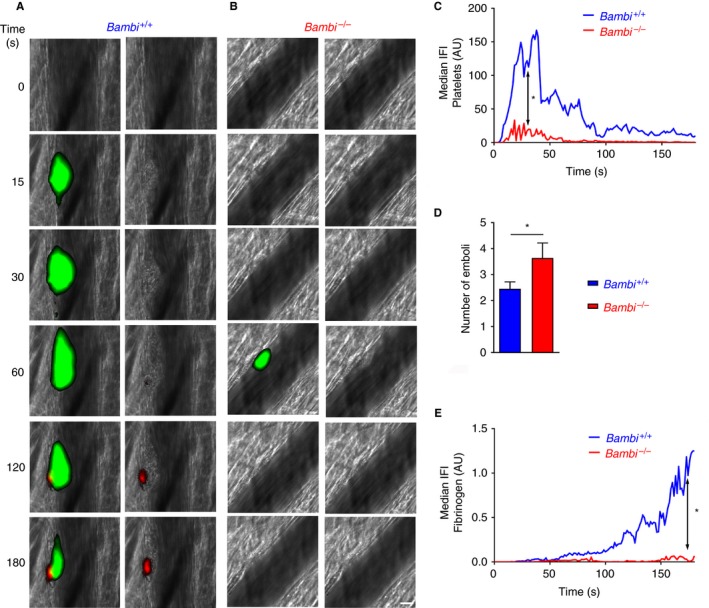
Lack of fibrin(ogen) accumulation in *Bambi* ^−/−^ thrombi after laser‐induced thrombosis model. A, B, Representative composite fluorescence images of platelets (green) and fibrin (red) (left panels) or fibrin only (right panels) with bright field images after the laser injury in cremaster arterioles of *Bambi* ^+/+^ and *Bambi* ^−/−^ mice. Platelets and fibrin were visualized by injecting rat anti‐GPIbβ‐DyLight 488 antibody and fibrinogen‐A647, respectively. Scale bar represents 10 μm. C, Graph represents median integrated fluorescence intensity (IFI) from platelets (AU, arbitrary unit) as a function of time after the injury (45 thrombi in seven *Bambi* ^+/+^ mice; 26 thrombi in four *Bambi* ^−/−^ mice). The area under the curve of fluorescence intensity over 180 s was analyzed using an unpaired Mann Whitney test; *P < .05. D, Number of emboli (>25% maximal thrombus size) counted during 3 min after laser injury. Results are shown as mean ± SEM. Statistical analysis was performed using unpaired *t* test, * *P* < .05. E, Graph represents median fibrin(ogen)‐IFI (AU) as a function of time after the laser injury. The area under the curve of fluorescence intensity over 180 s was analyzed using an unpaired Mann Whitney test, * *P* < .05. See also Movie S2 for better visualization of the differences in thrombus stability between *Bambi* ^+/+^ and *Bambi* ^−/−^ mice

We next hypothesized that *Bambi* deficiency may alter the anticoagulant function of the endothelium and therefore examined whether thrombin generation (as measured by fluorescent fibrin deposition) might be impaired in *Bambi‐*deficient mice. Following laser‐induced injury, thrombus instability in *Bambi* ^−/−^ mice, observed by decreased thrombus formation over time (Figure 3A‐C) and increased embolization events (Figure 3D), was accompanied by a large reduction (~80%) in fibrin accumulation over time (Figure 3E; Movie S2). This was not due to differences in endogenous fibrinogen levels as *Bambi* ^−/−^ mice exhibited normal plasma fibrinogen concentration (Figure S3).

**Figure 4:**
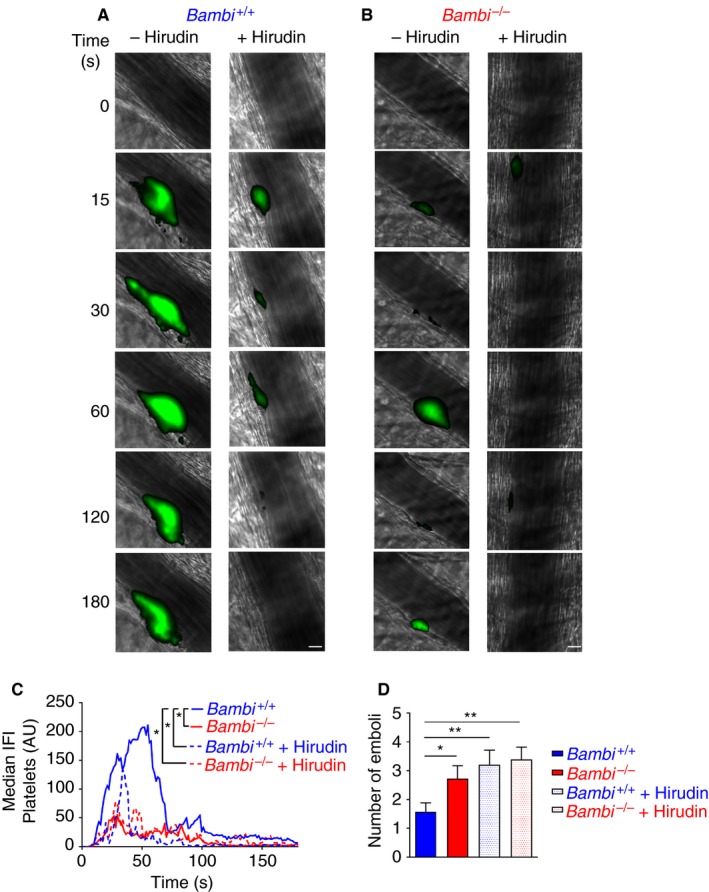
Hirudin‐injected *Bambi* ^+/+^ mice exhibit thrombus instability similar to *Bambi* ^−/−^ mice after laser‐induced thrombosis model. A, B, Representative composite fluorescence images of platelets (green) with bright field images after the laser injury in cremaster arterioles of *Bambi* ^+/+^ and *Bambi* ^−/−^ mice. Platelets were visualized by injecting rat anti‐GPIbβ‐DyLight 488 antibody. Scale bar represents 10 μm. C, D, For each mouse, four or five thrombi were performed prior and after hirudin injection via the jugular vein. *Bambi* ^+/+^ mice (*n* = 6) (‐hirudin *n* = 29 thrombi; +hirudin *n* = 20 thrombi); *Bambi* ^−/−^ mice *(n* = 7) (‐hirudin *n* = 37 thrombi; +hirudin *n* = 30 thrombi). C, Graph represents median integrated fluorescence intensity (IFI) from platelets (AU, arbitrary unit) as a function of time after the injury. The area under the curve of fluorescence intensity over 180 s was analyzed using an unpaired Mann Whitney test; * *P* < .05.D, Number of emboli (>25% maximal thrombus size) counted during 3 min after laser injury. Results are shown as mean ± SEM. Statistical analysis was performed using unpaired *t* test; * *P* < .05; ** *P* < .01. See Movie S3 for better visualization of the differences in thrombus stability between control group (wild‐type mice, no hirudin) and *Bambi* ^−/−^ or hirudin‐injected mice

To ascertain whether the reduced fibrin accumulation was due to inefficient thrombin generation we injected the potent thrombin inhibitor hirudin into mice prior to laser‐induced thrombosis. As previously shown in this model,^3^, ^44^, ^45^ injection of hirudin in wild‐type (*Bambi* ^+/+^) mice led to a reduction of platelet accumulation (Figure 4A‐C). Interestingly, inhibiting thrombin generation with hirudin in *Bambi* ^+/+^ mice appeared to phenocopy the thrombus instability of *Bambi* ^−/−^ mice (Figure 4D; Movie S3). Platelet thrombi formed initially (i.e. there was no difference in maximal platelet thrombus size observed in *Bambi* ^+/+^ mice in the presence of hirudin; data not shown) but because of the lack of fibrin accumulation thrombi were unstable. Interestingly, we saw no discernible effect of hirudin upon thrombus formation in *Bambi* ^−/−^ mice (Figure 4B‐D), supporting the contention that impaired fibrin deposition during thrombus formation is likely caused by reduced thrombin generation in *Bambi*‐deficient mice.

### 3.4 Tissue‐specific deletion of BAMBI in the endothelium leads to thrombus instability and lack of fibrin generation

Using chimeric *Bambi* ^−/−^ mice, we previously demonstrated that BAMBI in the vessel wall, rather than in the hematopoietic compartment, influenced thrombus formation.^34^ To explore the role of endothelial BAMBI (rather than other extravascular cells) in thrombus stability more specifically, we generated endothelium‐specific *Bambi*‐deficient mice (*Bambi* ^*flox/flox*^ *Tie2‐Cre* ^*+*^) using the Cre/Lox system and previously characterized *Tie2‐Cre* mice (Figure S4A–C).^46^, ^47^, ^48^ Unlike a proportion of *Bambi* ^−/−^ mice,^34^ all *Bambi* ^*flox/flox*^ *Tie2‐Cre* ^*+*^ mice were viable and pups were similar in weight when compared to *Bambi* ^flox/flox^ littermates. To evaluate the role of endothelial BAMBI in hemostasis, we performed tail bleeding assays. Whereas *Bambi* ^*flox/flox*^ *Tie2‐Cre* ^*+*^ mice exhibited a small increase in bleeding times compared to *Bambi* ^flox/flox^ mice, this was not significant (data not shown). Moreover, there was no significant difference in the extent of blood loss between the mice (Figure S4D).

**Figure 5:**
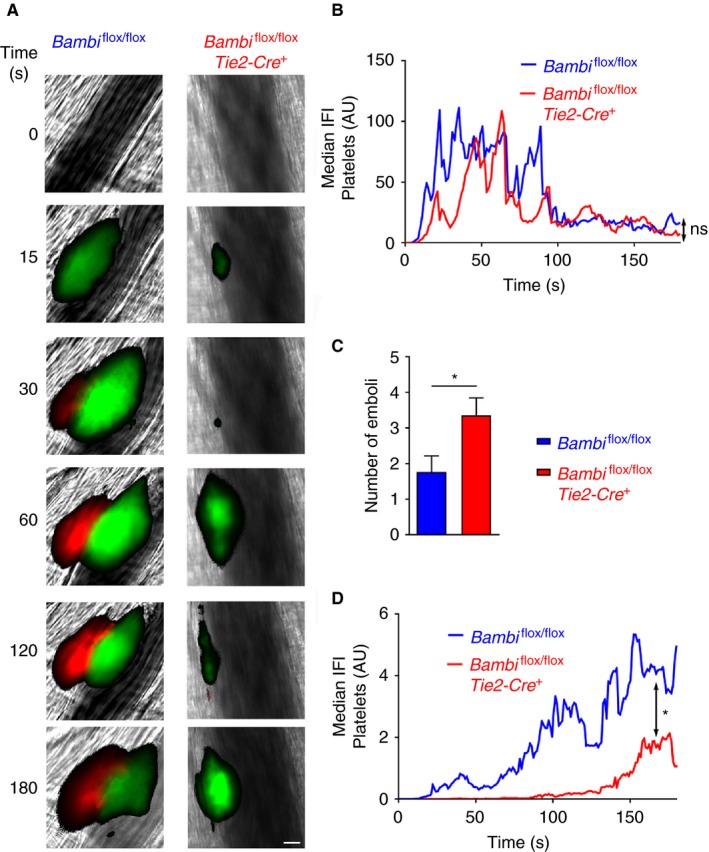
Endothelial BAMBI is responsible for the defect in thrombus stability in vivo. A, Representative composite fluorescence images of platelets (green) and fibrin (red) with bright field images after the laser injury in cremaster arterioles of *Bambi* ^flox/flox^ and *Bambi* ^flox/flox^ *Tie2‐Cre+* mice. Platelets and fibrin were visualized by injecting rat anti‐GPIbβ‐DyLight 488 antibody and fibrinogen‐A647, respectively. Scale bar represents 10 μm. B, Graph represents median integrated fluorescence intensity (IFI) from platelets (AU, arbitrary units) as a function of time after the injury (26 thrombi in four *Bambi* ^flox/flox^ mice; 28 thrombi in five *Bambi* ^flox/flox^ *Tie2‐Cre+* mice). The area under the curve of fluorescence intensity over 180 s was analyzed using an unpaired Mann Whitney test; ns: *P* > .05. C, Number of emboli (>25% maximal thrombus size) counted during 3 min after laser injury. Results are shown as mean ± SEM. Statistical analysis was performed using unpaired *t* test; * *P* < .05. D, Graph represents median fibrin(ogen)‐IFI (AU) as a function of time after the laser injury. The area under the curve of fluorescence intensity over 180 s was analyzed using an unpaired Mann Whitney test; * *P* < .05. See also Movie S4

To explore the role of endothelial BAMBI in thrombus formation, we performed the laser‐induced thrombosis model in *Bambi* ^*flox/flox*^ *Tie2‐Cre* ^*+*^ mice. There was no difference in platelet accumulation between *Bambi* ^*flox/flox*^ *Tie2‐Cre* ^*+*^ mice and *Bambi* ^flox/flox^ mice (Figure 5A,B), but there was a significant increase in thrombus embolization (Figure 5C; Movie S4). Thrombus instability was also accompanied with a decrease in fibrin accumulation (Figure 5D), as observed for *Bambi* ^−/−^ mice (Figure 3D). Collectively, these results suggest that deficiency of endothelial BAMBI influences both fibrin accumulation and thrombus stability after laser injury.

### 3.5 *Bambi* ^−/−^ mice exhibit increased TFPI and thrombomodulin expression levels

**Figure 6:**
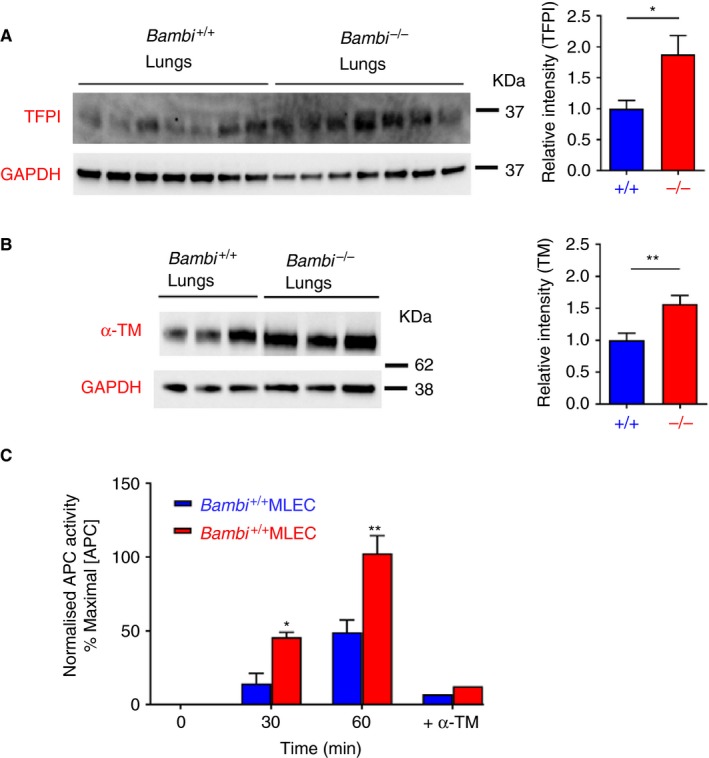
A, B, Increased thrombomodulin and TFPI expression levels in *Bambi* ^−/−^ mice. Representative Western blot of A, TFPI and B, thrombomodulin expression in *Bambi* ^+/+^ and *Bambi* ^−/−^ lung homogenates. Protein levels were quantified using Image Lab 5.2.1 software (Biorad), normalized against GAPDH controls and expressed as relative intensities (TFPI or TM/GAPDH). Values are given as mean ± SEM (*n* ≥ 7 mice per genotype from at least two Western blots). Statistical analysis was performed using unpaired Student *t* test; * *P* < .05; ** *P* < .01. Molecular weights from protein standards are indicated in kilodaltons on each Western blot. C, *Bambi* ^+/+^ and *Bambi* ^−/−^ MLEC were incubated with 100 nM human protein C in the presence of Ca^2+^ and activated by 2 nM thrombin. The reactions were stopped at the indicated times by addition of an excess of hirudin (100 nM).  The APC generation was quantified by determining the rate of chromogenic substrate S‐2366 (0.5 mM) cleavage at 405 nm and using an APC standard curve generated in parallel for each experiment (cf. Figure S5D). The APC generation was normaliszed to the number of cells and maximal APC activity for each assay. When indicated (+α‐TM), *Bambi* ^+/+^ and *Bambi* ^−/−^ MLEC were incubated for 30 min with a goat antimouse thrombomodulin antibody (50 nM) before addition of protein C and thrombin to the wells and APC activity was determined after 60 min. Values are given as mean ± SEM (*n* = two separate isolations). Statistical analysis was performed using two‐way ANOVA followed by Bonferroni posttests; * *P* < .05 ** *P* < .01. APC, activated protein C; GADPH, Glyceraldehyde 3‐phosphate dehydrogenase; MLEC, mouse lung endothelial cell; TFPI, tissue factor pathway inhibitor

Our results suggest that *Bambi*‐deficient EC are responsible for the reduced propensity to generate fibrin after vascular injury. We hypothesized that the thromboprotective phenotype observed in *Bambi*‐deficient mice could be due to increased anticoagulant function of the endothelium ‐ more specifically due to elevated TFPI function and/or APC generation. Although no significant differences in *Tfpi*,* Procr,* or *Thbd* mRNA levels (corresponding to TFPI, EPCR, and thrombomodulin genes, respectively) were detected in lungs or MLEC from *Bambi* ^−/−^ compared to *Bambi* ^+/+^ mice (data not shown), Western blot analysis of lung extracts revealed that both total TFPI and thrombomodulin levels were 60% to 80% increased in *Bambi* ^−/−^ mice compared to *Bambi* ^+/+^ mice (Figure 6A,B). Elevated thrombomodulin was also detected in MLEC and MBEC isolated from *Bambi* ^−/−^ mice compared to *Bambi* ^+/+^ mice (Figure S5). Thrombomodulin binds thrombin with high affinity and, in so doing, facilitates protein C activation. To assess the influence of elevated thrombomodulin levels on *Bambi* ^−/−^ MLEC functionally, we performed APC generation assays. Significant increase in APC generation after 30 and 60 min was detected in *Bambi* ^−/−^ MLEC compared to *Bambi* ^+/+^ MLEC (Figure 6C). The specificity of the assay for thrombomodulin was confirmed using an inhibitory antithrombomodulin polyclonal antibody.^39^ As shown in Figure 6C, blocking thrombomodulin decreased APC generation on both *Bambi* ^−/−^ and *Bambi* ^+/+^ MLEC by ~90%. These data demonstrate that elevated levels of thrombomodulin on EC from *Bambi* ^−/−^ mice can lead to increased propensity to generate APC.

### 3.6 Blocking TFPI and thrombomodulin function restores fibrin accumulation and thrombus stability in Bambi^−/−^ mice

To investigate the influence of elevated TFPI and thrombomodulin in *Bambi*‐deficient mice upon fibrin accumulation and thrombus stability in vivo, we performed the laser‐induced thrombosis model under conditions where each endogenous anticoagulant protein was blocked using inhibitory polyclonal antithrombomodulin and anti‐TFPI antibodies. Blocking thrombomodulin inhibited APC generation on cells in vitro (Figure 6C), and also using calibrated automated thrombography in the presence of recombinant murine soluble thrombomodulin (Figure S6A). The anti‐TFPI antibody inhibited murine TFPI function in FXa inhibition assays (

Figure S6B).

**Figure 7:**
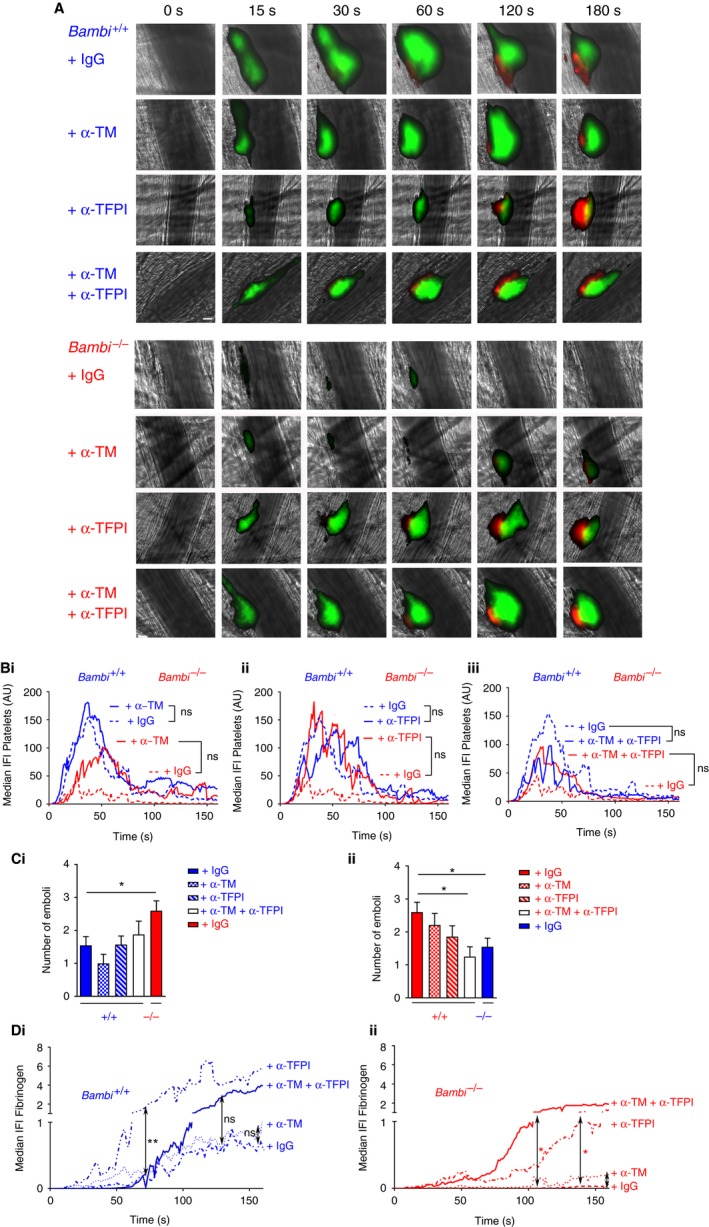
Inhibiting thrombomodulin and TFPI in vivo in *Bambi* ^−/−^ mice restores thrombus stability and fibrin generation in the laser‐induced thrombosis model. A, Representative composite fluorescence images of platelets (green) and fibrin (red) with bright field images after the laser injury in cremaster arterioles of *Bambi* ^+/+^ and *Bambi* ^−/−^ mice injected with control goat immunoglobulin G (IgG), goat anti mouse thrombomodulin antibody (α‐TM), goat antimouse TFPI (α‐TFPI), or a combination of both α‐TM and α‐TFPI. Platelets and fibrin were visualized by injecting rat anti‐GPIbβ‐DyLight 488 antibody and fibrinogen‐A647, respectively. Scale bar represents 10 μm. B, Graph represents median integrated fluorescence intensity (IFI) from platelets (AU, arbitrary unit) as a function of time after the injury. Thrombi from *Bambi* ^+/+^ and *Bambi* ^−/−^ mice injected with control goat IgG are overlaid with thrombi from mice injected with α‐TM (Bi), α‐TFPI (Bii), or a combination of both α‐TM and α‐TFPI antibodies (Biii) (43 thrombi in seven *Bambi* ^+/+^ mice (+ Goat IgG); 45 thrombi in six *Bambi* ^−/−^ mice (+ Goat IgG); 25 thrombi in three *Bambi* ^+/+^ mice (+ α‐TM); 27 thrombi in three *Bambi* ^−/−^ mice (+ α‐TM); 21 thrombi in two *Bambi* ^+/+^ mice (+ α‐TFPI); 28 thrombi in three *Bambi* ^−/−^ mice (+ α‐TFPI); 20 thrombi in two *Bambi* ^+/+^ mice (+ α‐TFPI + α‐TM); 20 thrombi in two *Bambi* ^−/−^ mice (+ α‐TFPI + α‐TM). The area under the curve of fluorescence intensity over 180 s was analyzed using an unpaired Mann Whitney test; ns: *P* > .05. C, Number of emboli (>25% maximal thrombus size) counted during 3 min after laser injury. Results are shown as mean ± SEM. Statistical analysis was performed using one‐way ANOVA with Dunnett's multiple comparisons test; versus *Bambi* ^+/+^ in Ci. or versus *Bambi* ^−/−^ in Cii. * *P* < .05. D, Graph represents fibrin(ogen)‐IFI (AU) as a function of time after the laser injury. Di. *Bambi* ^+/+^ mice injected with Goat IgGs, α‐TM, α‐TFPI, α‐TFPI and α‐TM antibodies. Dii. *Bambi* ^−/−^ mice injected with Goat IgGs, α‐TM, α‐TFPI, α‐TFPI and α‐TM antibodies. The area under the curve of fluorescence intensity over 180 s was analyzed using an unpaired Mann Whitney test; ** *P* < .01; * *P* < .05; ns: *P* > .05. See also Movies S5 and S6 for better visualization of the differences in thrombus stability between *Bambi* ^+/+^ and *Bambi* ^−/−^ mice. TFPI, tissue factor pathway inhibitor

Similar to earlier observations (Figures 1, 3, 4), *Bambi* ^−/−^ mice (injected with control goat IgG) exhibited a significant reduction in platelet accumulation over time (Figure 7A,B) and increased number of emboli (Figure 7C) compared to wild‐type animals (also injected with control goat IgG). As before, fibrin accumulation over time in *Bambi* ^−/−^ mice was also significantly attenuated (Figure 7A;Dii and Movie S5).

In wild‐type mice, inhibition of thrombomodulin alone had no significant effect upon platelet thrombus formation (Figure 7A, Bi), the frequency of embolization (Figure 7Ci), or fibrin accumulation after laser injury (Figure 7Di; Movie S6). In *Bambi* ^−/−^ mice, blocking thrombomodulin induced an increase in platelet accumulation (Figure 7Bi), reduced embolization (Figure 7Cii), and increased fibrin accumulation (Figure 7Dii; Movie S5) although it did not reach statistical significance.

Although blocking TFPI in wild‐type mice had no effect upon platelet thrombi or embolization (Figure 7Bii, Ci), this caused a significant increase in fibrin deposition (Figure 7Di; Movie S6), consistent with the primary role of TF in initiating coagulation in this model.^2^, ^44^, ^45^ A very similar effect was also seen in *Bambi* ^−/−^ mice (Figure 7Dii). Interestingly, simultaneous inhibition of both thrombomodulin and TFPI function in *Bambi* ^−/−^ mice significantly reduced embolization to similar levels to those observed in wild‐type mice (Figure 7Cii; Movies S5 and S6). Moreover, fibrin deposition was also normalized (Figure 7A;Dii). Collectively, these results suggest that loss of BAMBI influences fibrin formation and thrombus stability by altering the levels of thrombomodulin and TFPI on the endothelial surface.

## 4 DISCUSSION

We previously reported that lack of BAMBI reduces thrombus formation/stability in two different in vivo models of thrombosis.^34^ Using endothelial‐specific *Bambi*‐knockout mice and intravital imaging, we here confirm a role for endothelial BAMBI in the formation of a stable thrombus after vascular injury. Further, we show that the thrombus formation/stability defect in *Bambi*‐deficient mice is associated with defective fibrin accumulation. Our data also identify the contributions of elevated TFPI and thrombomodulin to the decreased thrombin generation and increased embolization seen in *Bambi* ^−/−^ mice.

Formation of a stable thrombus after vascular injury requires the concerted action of vessel wall components (e.g. extravascular tissue factor, subendothelial matrix, and activated EC), the coagulation system, platelets, and, as recently demonstrated, other cells including red blood cells and neutrophils.^2^, ^5^ The importance of neutrophil accumulation mediated by lymphocyte function‐associated antigen 1 (LFA‐1) expressed on neutrophils and ICAM‐1 expressed on EC in the laser‐induced thrombosis model of the cremaster arterioles has recently been demonstrated.^2^, ^49^ Using the same approach as Darbousset et al.^2^ we confirm the recruitment of neutrophils after the vascular injury in this thrombosis model; however, we detected normal accumulation of neutrophils/neutrophil‐derived particles in the laser‐induced thrombi of *Bambi* ^+/+^ and *Bambi* ^−/−^ mice (Figure 1). This suggested that EC present ICAM‐1 normally after activation,^50^ and consistent with this, no difference in the expression of several endothelial cell markers was detected in *Bambi* ^−/−^ MLEC (Figure 2). Once bound, neutrophils can enhance coagulation and promote thrombus formation.^49^ Conceivably, recruited *Bambi* ^−/−^ neutrophils may be defective in their activation, leading to reduced thrombin generation and fibrin. However, this was excluded as the hemostatic defect in *Bambi* ^−/−^ mice was phenocopied by the endothelial cell‐specific deletion of *Bambi* (Figure 5) and was also present in chimeric *Bambi* ^−/−^ mice reconstituted with wild‐type bone marrow cells.^34^ Similarly, although one cannot formally exclude a possible role for elevated fibrinolysis in thrombus instability caused by alterations in regulators associated with this pathway (e.g. Thrombin activatable fibrinolysis inhibitor, plasminogen activator inhibitor‐1, tissue plasminogen activator), based on the literature, it is unlikely they could be major contributors to the phenotype that we observed in *Bambi* ^−/−^ mice. Indeed, full deficiencies in these markers have not always been reported to protect against thrombosis depending on the model chosen in the study (arterial or venous thrombosis) and the vascular bed (carotid, mesenterium, femoral).^51^, ^52^, ^53^, ^54^ Moreover, in order for fibrinolysis to occur, fibrin needs to be deposited first at the site of injury before fibrinolysis is efficiently activated.

We next evaluated the influence of anticoagulant pathways in thrombus formation and stability in *Bambi* ^−/−^ mice. In the laser‐induced thrombosis model, endothelial activation results in increased ICAM‐1 and Lysosomal‐associated membrane protein 1 presentation.^50^, ^55^ Platelets (and leukocytes) are recruited at the site of injury, become activated by locally generated thrombin, which creates a core of activated platelets overlaid by a shell of P‐selectin negative platelets.^3^, ^8^ Fibrin is associated with the core of tightly packed P‐selectin‐positive platelets and extends into the vasculature 3, 8 but can also be detected away from the vascular injury on the activated endothelium itself.^6^ Strikingly, *Bambi* ^−/−^ and *Bambi* ^*flox/flox*^ *Tie2‐Cre* ^*+*^ mice were unable to accumulate fibrin efficiently or to sustain thrombus formation after vascular injury (Figures 3E and 5D). This may be unsurprising given the role of fibrin in thrombus stability.^56^, ^57^ However, it is also important to note that fibrin accumulation in this model can occur without appreciable platelet accumulation, as observed for example in *Par4* ^−/−^ mice.^45^ Another group has also shown that platelet accumulation at the site of vascular injury is not a prerequisite for fibrin deposition,^6^ supporting the contention that altered platelet accumulation is unlikely to be responsible for the reduced ability of *Bambi* ^−/−^ mice to generate fibrin.

Despite the clear defect in fibrin accumulation and increased thrombus instability, platelet accumulation over time after the vascular injury in *Bambi* ^*flox/flox*^ *Tie2‐Cre* ^*+*^ mice was not statistically different from that in *Bambi* ^*flox/flox*^ mice (Figure 5B), suggesting that thrombi are more likely reforming in *Bambi* ^*flox/flox*^ *Tie2‐Cre* ^*+*^ mice after embolization. Interestingly, in contrast to *Bambi* ^−/−^ mice, *Bambi* ^*flox/flox*^ *Tie2‐Cre* ^*+*^ mice had also normal hemostasis/tail bleeding (Figure S4), exhibited no difference in weight compared to littermates, and were all viable. Our data therefore point to an important role for endothelial BAMBI in fibrin deposition/thrombus stability but imply that the phenotypic differences observed between the constitutive and conditional *Bambi* knockout mice are manifest through roles for BAMBI in cellular compartments other than the endothelium.

Injection of hirudin in *Bambi* ^+/+^ mice had minimal effect on the maximal platelet thrombus size (i.e. platelet accumulation) reached within 40 to 60 s post injury (data not shown), but had significant effect upon overall thrombus formation (Figure 4C) because of the reduction of fibrin deposition, which was associated with decreased thrombus stability (Figure 4D). These effects of hirudin on thrombus formation are consistent with several reports,^3^, ^4^, ^45^, ^58^ as well as its minimal effect on maximal thrombus size.^58^ Interestingly, the kinetics of thrombus formation in hirudin‐injected *Bambi* ^+/+^ mice closely resembled those observed in *Bambi* ^−/−^ mice. Moreover, injection of hirudin into *Bambi* ^−/−^ mice did not further diminish thrombus formation or further increase the number of emboli. These results strongly suggest that the lack of fibrin accumulation in *Bambi* ^−/−^ mice is the major cause of the thrombus instability, and that the reason for reduced fibrin in the thrombus is most likely due to lower levels of thrombin generation after the injury. Naturally, lower levels of thrombin generated at the site of injury would also impact upon platelet activation and exacerbate the thrombus instability phenotype in *Bambi* ^−/−^ mice.

The increase in thrombomodulin levels on *Bambi* ^−/−^ MLEC was associated with increased ability to generate APC (Figure 6). In vivo inhibition of thrombomodulin in *Bambi* ^−/−^ mice moderately improved fibrin deposition, platelet accumulation, and thrombus stability (Figure 7Bi;Cii;Dii). The TFPI blockade in *Bambi* ^−/−^ mice had a similar influence upon platelet accumulation and thrombus stability as blocking thrombomodulin (Figure 7Bii, Cii). However, fibrin accumulation was significantly increased (Figure 7Dii). Interestingly, inhibition of thrombomodulin in *Bambi* ^+/+^ had little effect on fibrin accumulation, whereas TFPI inhibition led to a dramatic increase in fibrin (Figure 7Di). Our results therefore suggest that the importance of TFPI in regulating coagulation in the laser injury thrombosis model exceeds that of thrombomodulin. This is consistent with the well‐documented role of TF in this model.^2^, ^44^, ^45^ This may also be compatible with the role of thrombomodulin in limiting the spread of the hemostatic plug, rather than diminishing thrombin generation within the core of the thrombus.

Shortening of vessel occlusion times and increased thrombus volumes have been reported in mice variably (rather than completely) deficient in TFPI and thrombomodulin using different thrombosis models;^59^, ^60^, ^61^, ^62^, ^63^ however, this is the first report evaluating the importance of these proteins in the laser‐induced thrombosis model of the cremaster muscle arterioles. Ellery et al. showed no effect upon fibrin accumulation in *Tfpi* ^−/−^ *Par4* ^−/−^ compared to *Par4* ^−/−^ mice using a venous electrolytic injury model.^63^ There was also no discernible contribution of platelet TFPI to fibrin generation in this model 62 unless combined with *FVIII* deficiency.^61^ As suggested by Ellery et al^63^ the electrolytic injury model may not be particularly sensitive to changes in endothelial TFPI levels because of the nature of the injury, which may be less dependent upon TF for fibrin deposition than in laser‐induced thrombus formation (Figure 7D).

Inhibition of thrombomodulin or TFPI alone in *Bambi* ^−/−^ mice was not sufficient to restore thrombus stability, whereas combined targeting of these anticoagulant proteins in these mice led to further increase in fibrin deposition in addition to a decrease in embolization events (Figure 7Ciii,Dii). Mechanistically, elevated TFPI levels on the endothelium could contribute to inefficient thrombin generation, particularly in the settings of mild injuries that are dependent upon TF. Once coagulation is allowed to proceed, elevated thrombomodulin levels promoting protein C activation could further accentuate it, leading to lack of fibrin accumulation in *Bambi* ^−/−^ mice. The reason why blocking thrombomodulin alone did not exert a stronger effect may be linked to the dependency of this pathway upon thrombin. When thrombomodulin alone is inhibited, the elevated TFPI levels do not permit sufficient thrombin generation to activate this pathway appreciably. Only when TFPI is inhibited does this provide increased thrombin that enables the contribution of the protein C pathway to be detected in *Bambi* ^−/−^ mice. Together, these data suggest that reduced fibrin deposition after injury in *Bambi*‐deficient mice is linked to higher levels of both thrombomodulin and TFPI on the endothelial cell surface. Of note, we did not detect any difference in expression levels of plasma TFPI (TFPIγ) in *Bambi* ^−/−^ mice (Figure S7).

In conclusion, we demonstrate that BAMBI influences the phenotype of the endothelium and deficiency from these cells precipitates thrombus instability due to reduced fibrin deposition. Our data suggest that BAMBI mediates its effect via modulation of the natural anticoagulant function of the endothelium. As we found no discernible/major differences in *Tfpi* or *Thbd* mRNA levels in *Bambi* ^−/−^ mice, we hypothesize that BAMBI may influence cell surface thrombomodulin and TFPI levels through modulation of cell surface shedding and/or internalization, which is the focus of ongoing studies. It remains unclear how BAMBI exerts its function at a molecular level, whether via TGFβ/BMP/activin‐dependent or TGFβ/BMP/activin‐independent mechanisms. Understanding how and when BAMBI interacts with both extracellular and intracellular binding partners will help define its physiological role and how this influences endothelial function.

## AUTHOR CONTRIBUTIONS

James T. B. Crawley designed the research and wrote the paper; Argita Zalli, James H. Monkman, and Anastasis Petri performed experiments and analyzed data; David A. Lane designed the research and revised the manuscript; Josefin Ahnström designed the research, analyzed data, and revised the manuscript; Isabelle I. Salles‐Crawley designed and performed experiments, analyzed data, and wrote the paper.

## CONFLICT OF INTEREST

The authors state that they have no conflict of interest.
