# Supplementary material for: Defective fibrin deposition and thrombus stability in Bambi −/− mice are mediated by elevated anticoagulant function
Source: J Thromb Haemost. 2019 Aug 26;17(11):1935–49. doi: 10.1111/jth.14593 (PMC6899896; doi:10.1111/jth.14593)
Supplement: Supplementary file 1 [file JTH-17-1935-s007.pdf]

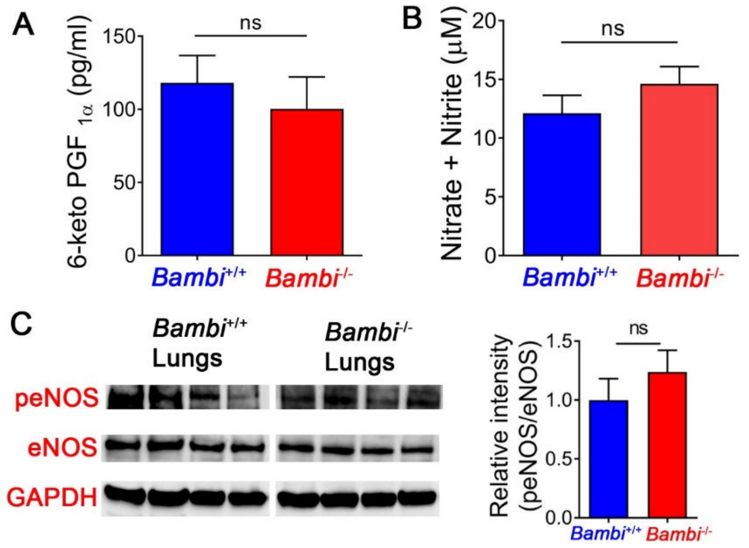

Fig. S1. Normal plasma prostacyclin and nitric oxide levels in *Bambi*$^{-/-}$ mice. Mouse plasma was collected from *Bambi*$^{-/-}$ mice and littermates controls for measurement of the stable prostaglandin I2 (prostacyclin) derivative, 6-keto-PGF$_{1\alpha}$ (A) and nitrates and nitrites (nitric oxide, B) as described in supplemental methods. The data represent the mean ± SEM (n≥5 for each genotype). Statistical analysis was performed using unpaired student *t*-test (p>0.05). (C) Representative western blot of phosphorylated eNOS and eNOS in lung extracts from *Bambi*$^{+/+}$ and *Bambi*$^{-/-}$ mice (n=4). Protein levels were quantified using Image Lab 5.2.1 software (Biorad), normalised against GAPDH controls and expressed as relative intensity (peNOS/eNOS). Values are given as mean ± SEM from 2 western blots (n=4 for each genotype; right graph). Statistical analysis was performed using unpaired student *t*-test: non-significant (ns), p>0.05.

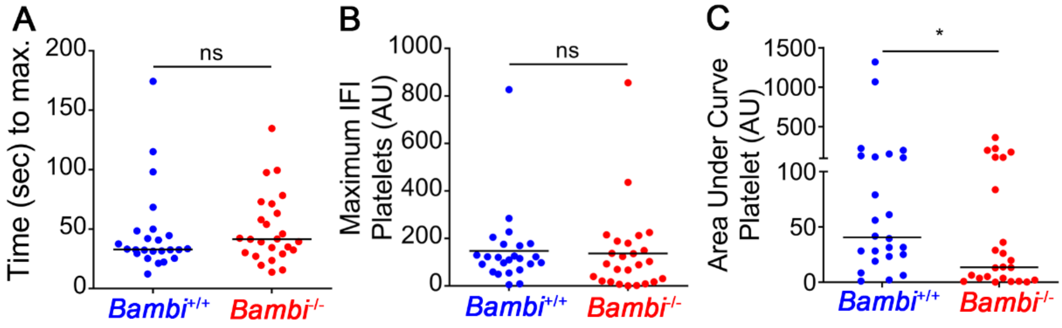

Fig. S2. Initial phases of thrombus formation are similar in *Bambi*$^{-/-}$ compared to *Bambi*$^{+/+}$ mice. Mice were subjected to the laser-induced thrombosis model as detailed in Figure 1. Distribution of the time to maximal thrombus size (A) and maximal thrombus size expressed in IFI platelet arbitrary units (AU) (B). (C) Graph showing the area under curve values from the platelet IFI vs time from individual thrombus. Each symbol represents one thrombus (23-25 thrombi in 3 mice for each genotype). Horizontal lines intersecting the data set represent the median. Statistical analysis was performed using Mann Whitney test: non-significant (ns): p>0.05; *p<0.05.

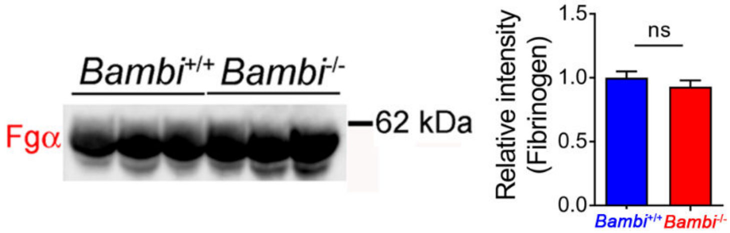

Fig. S3. Normal plasma fibrinogen levels in *Bambi*$^{-/-}$ animals Plasma from *Bambi*$^{+/+}$ and *Bambi*$^{-/-}$ mice (n=3) were collected, diluted in TBS buffer (1/100) and loaded on a 4-12% gel. Membranes were blotted and probed with a polyclonal anti-human fibrinogen-α antibody (representative western blot, left). Fibrinogen levels were quantified using Image Lab 5.2.1 software (Biorad) and normalized against total protein levels (right; mean ± SEM). Statistical analysis was performed using unpaired student *t*-test: non-significant (ns), $p>0.05$.

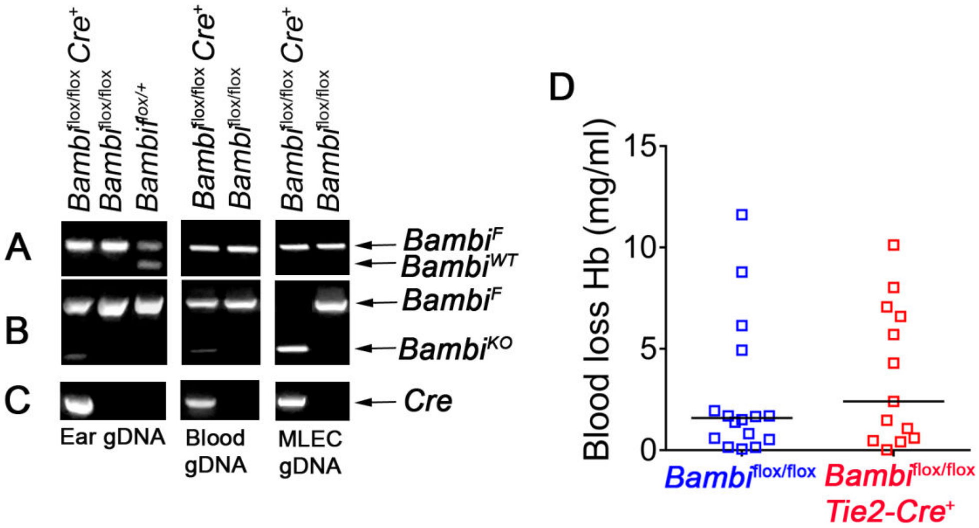

Fig. S4. Successful deletion of *Bambi* allele in endothelial cells. (A-C) PCR was performed on genomic DNA (gDNA) isolated from ear biopsies, blood or MLEC from *Bambi*$^{flox/flox}$, *Bambi*$^{flox/+}$ and *Bambi*$^{flox/flox}$ *Tie2-Cre*$^{+}$ mice as described in materials and methods. (A) PCR leading to amplification of the *Bambi* wild-type allele (225bp) and *Bambi*^{flox} allele (336bp) using F2 and R2 primers; (B) PCR leading to amplification of the *Bambi*^{flox} allele (>1kbp; top) and the *Bambi*-deleted allele (316bp) using F2 and R4 primers; (C) PCR leading to amplification of the *Cre* transgene (720 bp). (D) *Bambi*$^{flox/flox}$ (n=16) and *Bambi*$^{flox/flox}$ *Tie2-Cre*$^{+}$ mice (n=13) were subjected to the tail-bleeding assay. Blood was collected in PBS for the initial 10 min and blood loss was determined by quantification of haemoglobin (Hb) content in each collected sample. Each symbol represents one animal. Horizontal lines intersecting data sets represent the median. Statistical analysis was performed using Mann Whitney test: non-significant (ns), $p>0.05$.

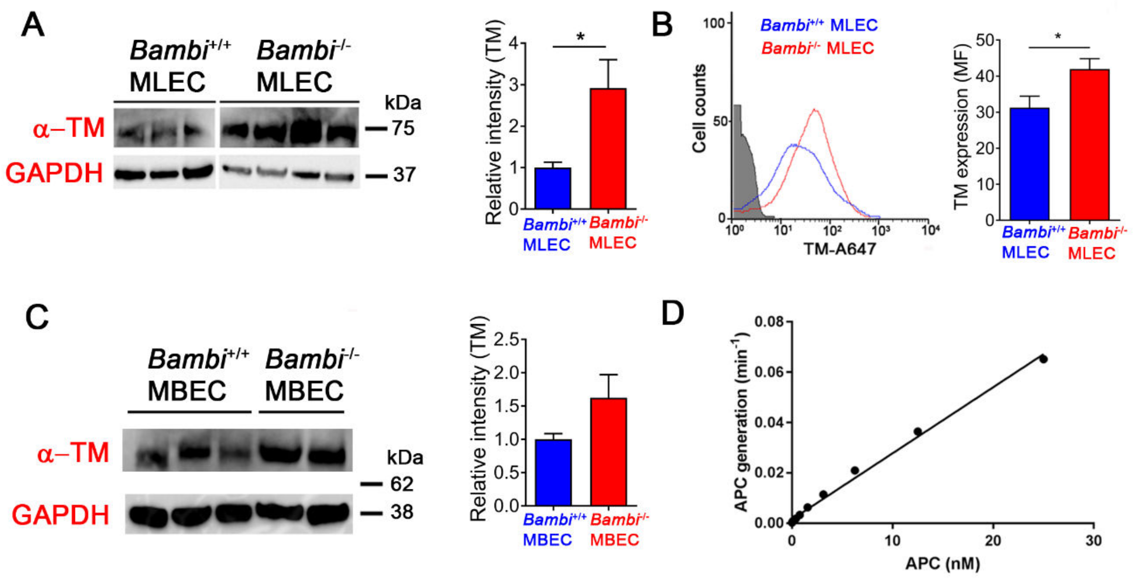

Fig. S5. Increased thrombomodulin levels in endothelial cells isolated from *Bambi*$^{-/-}$ mice. (A) Representative western blot of thrombomodulin (TM) expression in endothelial cells isolated from lungs of *Bambi*$^{+/+}$ and *Bambi*$^{-/-}$ mice (MLEC). Protein levels were quantified using Image Lab 5.2.1 software (Biorad), normalised against GAPDH controls and expressed as relative (TM/GAPDH) intensities. Values are given as mean ± SEM (n≥12 from at least 4 separate isolations and 5 western blots; right graphs). Molecular weights from protein standards are indicated in kDa on each western blot. Statistical analysis was performed using unpaired student *t*-test with Welch's correction, *p<0.05. (B) Representative flow cytometry analysis of *Bambi*$^{+/+}$ MLEC and *Bambi*$^{-/-}$ MLEC for thrombomodulin (left). Results are given as mean fluorescence intensities (MFI) ± SEM (n≥10 from 3 separate isolations; passages 2-8). Statistical analysis was performed using unpaired student t test, p<0.05. (C) Representative western blot of thrombomodulin (TM) expression in endothelial cells isolated from brains of *Bambi*$^{+/+}$ and *Bambi*$^{-/-}$ mice (MBEC; 5 mice per group). Protein levels were quantified using Image Lab 5.2.1 software (Biorad), normalised against GAPDH controls and expressed as relative intensities (TM/GAPDH). Values are given as mean ± SEM: *Bambi*$^{+/+}$ MBEC (n=3 separate isolations); *Bambi*$^{-/-}$ MBEC (n=2 separate isolations). Molecular weights from protein standards are indicated in kDa on each western blot. Statistical analysis was performed using unpaired student *t*-test with Welch's correction: non-significant (ns), p>0.05 (right graphs). (D) APC standard curve generated for each experiment used to determine APC activity in *Bambi*$^{+/+}$ MLEC and *Bambi*$^{-/-}$ MLEC. APC generation was quantified by determining the rate of chromogenic substrate S-2366 (0.5mM) cleavage at 405nm (cf. Fig. 6C).

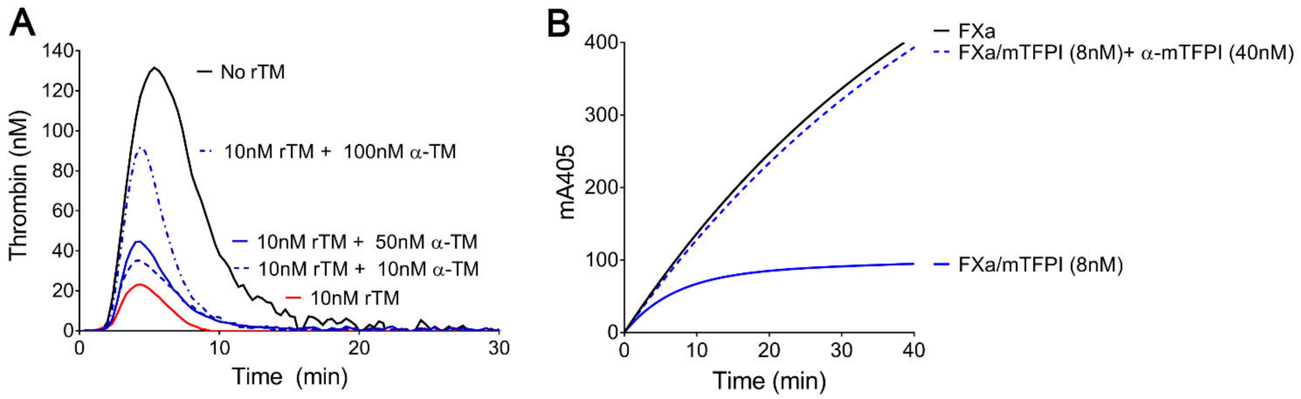

Fig.S6. Inhibition of thrombomodulin and TFPI function by polyclonal anti-mouse antibodies. (A) Thrombin generation was measured in human plasma supplemented with 4µM phospholipids, 4pM TF, 10nM recombinant mouse thrombomodulin (rTM) and increasing concentrations of polyclonal goat anti-mouse thrombomodulin (α-TM). Comparing the peaks of thrombin generation, adding 10nM rTM lowered thrombin generation by 82% (—); inhibiting TM function by adding 100nM α-TM (— ·) increased thrombin generation by 4-fold to reach 70% of control (—). (B) FXa (0.5nM) activity was measured in pure-component FXa inhibition assay through cleavage of S-2765 (200µM) in the presence of 25µM phospholipids, the presence and absence of recombinant mouse TFPI (mTFPI; 8nM), and 40nM of polyclonal goat anti-mouse TFPI (α-TFPI) as indicated. Addition of mTFPI to the reaction led to a decrease in FXa activity by 76% (—) compared to the control (—). Addition of the α-TFPI antibody fully restored FXa activity (--) demonstrating that TFPI function was fully inhibited by the addition of the antibody. Results from a representative experiment are shown (n=3).

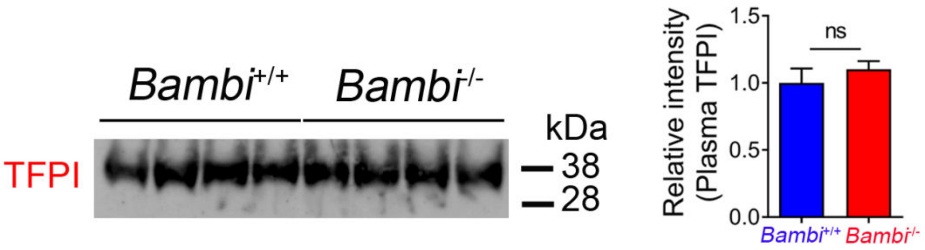

Fig. S7. Normal plasma TFPI levels in *Bambi*$^{-/-}$ animals Plasma from *Bambi*$^{+/+}$ and *Bambi*$^{-/-}$ mice (n=4) were collected, diluted in TBS buffer (1/100) and loaded on a 4-12% gel. Membranes were blotted and probed with a polyclonal anti-mouse TFPI antibody (representative western blot, left). Molecular weights from protein standards are indicated in kDa. TFPI levels were quantified using Image Lab 5.2.1 software (Biorad) (right; mean ± SEM). Statistical analysis was performed using unpaired student *t*-test: non-significant (ns), p>0.05.

Movie S1. Laser-induced thrombus formation in a *Bambi*$^{+/+}$ and *Bambi*$^{-/-}$ mouse: platelet and neutrophil recruitment to the vascular injury. Representative video of fluorescently-labeled platelets (green) and neutrophils (red) accumulating at the site of laser-induced injury in a cremaster muscle arteriole of a *Bambi*$^{+/+}$ and *Bambi*$^{-/-}$ mouse. Thrombus formation was studied using a combination of brightfield and fluorescence microscopy. A yellow color is seen when platelets and neutrophils are detected in the same thrombus region. Results are presented in Fig. 1. A timer is shown in the top left corner (hh:mm:ss:000) and a 10 µm scale bar in the bottom left corner.

Movie S2. Laser-induced thrombus formation in a *Bambi*$^{+/+}$ and *Bambi*$^{-/-}$ mouse: platelet and fibrin accumulation to the vascular injury. Representative video of fluorescently-labeled platelets (green) and fibrin(ogen) (red) accumulating at the site of laser-induced injury in a cremaster muscle arteriole of a

Bambi$^{+/+}$ and *Bambi*$^{-/-}$ mouse. Thrombus formation was studied using a combination of brightfield and fluorescence microscopy. A yellow color is seen when platelets and fibrin are detected in the same thrombus region. Results are presented in Fig. 3. A timer is shown in the top left corner (hh:mm:ss:000) and a 10 µm scale bar in the bottom left corner.

Movie S3. Laser-induced thrombus formation in a *Bambi*$^{+/+}$ and *Bambi*$^{-/-}$ mouse: the effect of hirudin on thrombus stability. Representative video of fluorescently-labeled platelets (green) accumulating at the site of laser-induced injury in a cremaster muscle arteriole of a *Bambi*$^{+/+}$ and *Bambi*$^{-/-}$ mouse injected or not with hirudin. Thrombus formation was studied using a combination of brightfield and fluorescence microscopy. Results are presented in Fig. 4. A timer is shown in the top left corner (hh:mm:ss:000) and a 10 µm scale bar in the bottom left corner.

Movie S4. Laser-induced thrombus formation in a *Bambi*$^{flox/flox}$ and *Bambi*$^{flox/flox}$ *Tie2-Cre*$^{+}$ mouse: platelet and fibrin accumulation to the vascular injury. Representative video of fluorescently-labeled platelets (green) and fibrin(ogen) (red) accumulating at the site of laser-induced injury in a cremaster muscle arteriole of a *Bambi*$^{+/+}$ and *Bambi*$^{-/-}$ mouse. Thrombus formation was studied using a combination of brightfield and fluorescence microscopy. A yellow color is seen when platelets and fibrin are detected in the same thrombus region. Results are presented in Fig. 5. A timer is shown in the top left corner (hh:mm:ss:000) and a 10 µm scale bar in the bottom left corner.

Movie S5. Laser-induced thrombus formation in a *Bambi*$^{-/-}$ mouse: the effect of inhibiting thrombomodulin, TFPI or both in platelet and fibrin accumulation. Representative video of fluorescently-labeled platelets (green) and fibrin(ogen) (red) accumulating at the site of laser-induced injury in a cremaster muscle arteriole of a *Bambi*$^{-/-}$ mouse. When indicated, mice were injected with the following antibodies: control goat IgG (top left), goat anti-mouse thrombomodulin (α-TM; top right), goat anti-mouse TFPI (α-TFPI; bottom left), or both (α-TM + α-TFPI; bottom right). Thrombus formation was studied using a combination of brightfield and fluorescence microscopy. A yellow color is seen when platelets and fibrin are detected in the same thrombus region. Results are presented in Fig. 7. A timer is shown in the top left corner (hh:mm:ss:000) and a 10 µm scale bar in the bottom left corner.

Movie S6. Laser-induced thrombus formation in a *Bambi*$^{+/+}$ mouse: the effect of inhibiting thrombomodulin, TFPI or both in platelet and fibrin accumulation. Representative video of fluorescently-labeled platelets (green) and fibrin(ogen) (red) accumulating at the site of laser-induced injury in a cremaster muscle arteriole of a *Bambi*$^{+/+}$ mouse. When indicated, mice were injected with the following antibodies: control goat IgG (top left), goat anti-mouse thrombomodulin (α-TM; top right), goat anti-mouse TFPI (α-TFPI; bottom left), or both (α-TM + α-TFPI; bottom right). Thrombus formation was studied using a combination of brightfield and fluorescence microscopy. A yellow color is seen when platelets and fibrin are detected in the same thrombus region. Results are presented in Fig. 7. A timer is shown in the top left corner (hh:mm:ss:000) and a 10 µm scale bar in the bottom left corner.
